# Authors’ Response to Peer Reviews of “Left Ventricular Outflow Tract Obstruction in Patients Treated With Milrinone for Cerebral Vasospasm: Case Report and Literature Review”

**DOI:** 10.2196/37114

**Published:** 2022-04-13

**Authors:** Charles Baulier, Marc Lessert, Jean-Louis Chauvet, Pauline Garel, Alexandre Bergis, Julie Burdeau, Thomas Clavier

**Affiliations:** 1 Anesthesia and Intensive Care Department Rouen University Hospital Rouen France; 2 Intensive Care Unit Elbeuf General Hospital Elbeuf France; 3 Cardiology Department Rouen University Hospital Rouen France

**Keywords:** ventricular outflow obstruction, subarachnoid hemorrhage, vasospasm, intracranial, milrinone, hemorrhage, neurosurgery, neurology, surgery, pharmaceutical

*This is the authors’ response to peer review reports of “Left Ventricular Outflow Tract Obstruction in Patients Treated With Milrinone for Cerebral Vasospasm: Case Report and Literature Review.”*

## Round 1 Review

### Reviewer AA [1]

#### General Comments

This is an interesting paper [2]. Overall, the information is well presented. That said, there are some areas that need improvements.

#### Specific Comments

##### Major Comments

1. This type of left ventricular outflow tract obstruction (LVOTO) should be addressed as dynamic LVOTO.
  Response to the first comment: We will insist more on the difference between the fixed obstruction and the dynamic obstruction. Fixed LVOTO is due to anatomical features (excessive mitral tissue, mitro-aortic angle less than 120°, septal hypertrophy). Dynamic LVOTO is due to the addition of predisposing factors (decreased preload and afterload, increased inotropism).
2. LVOTO per se should be briefly explained in the “Introduction” for the benefit of noncardiology readers: what LVOTO means, types of LVOTO such as fixed and dynamic, and a brief and simple explanation of dynamic LVOTO.
  Response to the second comment: As you say, our introduction will include a paragraph briefly explaining LVOTO, its definition, and its mechanisms. It is a complex hemodynamic phenomenon in itself, and its description must be rigorous. To describe it exhaustively in the introduction would risk making this part of the manuscript too long, but a complete description will stay in a dedicated part of the manuscript.
3. For the second patient, pages 7 and 8 state “In view of the hemodynamic improvement and the good neurological course, treatment with milrinone was continued at the same dose.” It looks like a repeat echo was done only after stopping milrinone. Was any echo repeated after hemodynamic improvement while the patient was continued on milrinone? How did you come to the conclusion that LVOTO is because of milrinone? He also had meningitis/sepsislike state (mentioned as an inflammatory syndrome in the manuscript), which in itself could predispose to LVOTO. Additionally, LVOTO can occur postoperatively after noncardiac surgery in patients with no known heart disease, and this patient also had a surgical procedure in the form of ventricular drain. These aspects are well discussed in reference 16 of the manuscript.
  Response to the second comment: Response to the third comment: For us, it is certain that the occurrence of LVOTO in our second patient was due to the addition of two predisposing factors: the use of milrinone and hypovolemia induced by sepsis. An echocardiogram in the presence of only one of the two elements could have made it possible to evaluate the dominance of one over the other. It is known that LVOTO is found in about 20% of patients in septic shock. However, this patient presented with a neurological infection with a state of relative hypovolemia, not being in shock. The systolic murmur persisted after punctual use of crystalloids, and only disappeared when the milrinone infusion was stopped. These elements support, in our opinion, the hypothesis that the use of milrinone was the main and triggering mechanism, sepsis being only an aggravating factor.
4. What is the explanation for unilateral left sided pulmonary edema for the first patient (as pulmonary edema is mostly bilateral in heart failure).
  Response to the fourth comment: Mitral regurgitation associated with LVOTO is most often eccentric and travels to the left pulmonary veins, resulting in unilateral acute pulmonary edema. We will incorporate this into the manuscript.

##### Minor Comments

1. The authors mention vasospasm was diagnosed using a computerized tomography (CT) scan. Plain CT scans are not used for the diagnosis of vasospasm, and they need to be more specific as to how vasospasm was diagnosed (eg, CT angio, Doppler study, or perfusion scan).
  Response to the fifth comment: We will specify in the manuscript that these were angio-CT

### Reviewer AN [3]

This paper deals with a rare event on the occurrence of left ventricular outflow obstruction in a patient treated with milrinone for vasospasm following an aneurysmal bleed.

#### Major Comments

1. The rationale for radioembolization of aneurysms needs to be elaborated.
  Response: The treatment option chosen was the most suitable for the patient, after CT analysis of the aneurysm characteristics and a discussion between a neuroradiologist and neurosurgeon.
2. The probable differential diagnosis of stunned myocardium syndrome in the acute phase needs to be mentioned.
  Response: We will include a brief description of neurogenic myocardial stunning in the manuscript. It is a not-so-rare complication of aneurysmal meningeal hemorrhage. This occurs in the acute phase of subarachnoid hemorrhage. Unlike LVOTO, beta receptor overstimulation results in myocardial sideration. The common feature of the two conditions is the worsening with the use of catecholamines.

## Round 2 Review

### Reviewer AA

#### Specific Comments

##### Major Comments

1. Page 6, lines 10-12 states “Although milrinone was administered at a constant dosage of 1 μg/kg/min, the clinical presentation led to find the origin of the shock: an accidental bolus of a milrinone due to a plication of the central venous catheter line during nursing care”. Would recommend clarifying this statement and explaining what exactly you mean by plication and how it resulted in an accidental bolus of milrinone.
  Response: The main hypothesis is that the catheter plicated when the patient was placed in a sitting position, the electric syringe did not stop, and the bolus of milrinone occurred when the obstruction was removed.
2. Bedside limited echocardiography is a routine practice to check the effect of various interventions in the intensive care unit. Therefore, it should be explained why echocardiography was not repeated in the second patient after hemodynamic improvement while the patient was continued on milrinone. Just relying on “systolic murmur” is not enough. Moreover, a murmur is also not described in detail. The murmur description should include intensity, quality, radiation, timing (pan systolic/short systolic), etc.
  Response: We have added a more specific description of the murmur in the manuscript. There was no follow-up echocardiography before discontinuation of milrinone therapy because the patient’s hemodynamic status was subsequently stable.
3. “Mitral regurgitation associated with LVOTO is most often eccentric, and travels to the left pulmonary veins, resulting in unilateral acute pulmonary edema in this patient.” Please provide a reference for this.
  Response: We have added a bibliographic reference for this.

## Abbreviations

CT: computerized tomography
LVOTO: left ventricular outflow tract obstruction
